# An Image-Analysis-Based Method for the Prediction of Recombinant Protein Fiber Tensile Strength

**DOI:** 10.3390/ma15030708

**Published:** 2022-01-18

**Authors:** Fredrik G. Bäcklund, Benjamin Schmuck, Gisele H. B. Miranda, Gabriele Greco, Nicola M. Pugno, Jesper Rydén, Anna Rising

**Affiliations:** 1 Department of Biosciences and Nutrition, Karolinska Institutet, Neo, 14186 Huddinge, Sweden; benjamin.schmuck@ki.se (B.S.); anna.rising@ki.se (A.R.); 2 Department of Anatomy, Physiology and Biochemistry, Swedish University of Agricultural Sciences, 75007 Uppsala, Sweden; 3 Division of Computational Science and Technology, KTH Royal Institute of Technology, 10044 Stockholm, Sweden; gmirand@kth.se; 4 BioImage Informatics Facility, Science for Life Laboratory, 17165 Solna, Sweden; 5 Laboratory for Bioinspired, Bionic, Nano, Meta, Materials & Mechanics, Department of Civil, Environmental and Mechanical Engineering, University of Trento, Via Mesiano, 77, 38123 Trento, Italy; gabriele.greco-2@unitn.it (G.G.); nicola.pugno@unitn.it (N.M.P.); 6 School of Engineering and Materials Science, Queen Mary University of London, Mile End Road, London E1 4NS, UK; 7 Department of Energy and Technology, Swedish University of Agricultural Sciences, 75007 Uppsala, Sweden; jesper.ryden@slu.se

**Keywords:** spider silk, protein fibers, image analysis, structure–function relationship, prediction, mechanical properties

## Abstract

Silk fibers derived from the cocoon of silk moths and the wide range of silks produced by spiders exhibit an array of features, such as extraordinary tensile strength, elasticity, and adhesive properties. The functional features and mechanical properties can be derived from the structural composition and organization of the silk fibers. Artificial recombinant protein fibers based on engineered spider silk proteins have been successfully made previously and represent a promising way towards the large-scale production of fibers with predesigned features. However, for the production and use of protein fibers, there is a need for reliable objective quality control procedures that could be automated and that do not destroy the fibers in the process. Furthermore, there is still a lack of understanding the specifics of how the structural composition and organization relate to the ultimate function of silk-like fibers. In this study, we develop a new method for the categorization of protein fibers that enabled a highly accurate prediction of fiber tensile strength. Based on the use of a common light microscope equipped with polarizers together with image analysis for the precise determination of fiber morphology and optical properties, this represents an easy-to-use, objective non-destructive quality control process for protein fiber manufacturing and provides further insights into the link between the supramolecular organization and mechanical functionality of protein fibers.

## 1. Introduction

Protein fibers, such as those made by spiders or silk moths, have been found to present remarkable properties, and significant research efforts both experimentally and with computer modeling have been spent on elucidating the underlying mechanics of these spectacular materials [1,2,3,4,5,6,7,8,9,10,11,12,13,14,15]. As reviewed previously, natural silk has numerous and diverse applications [16,17], ranging from, e.g., medicine [18,19,20] to photonics [21,22]. Although native spider silk fibers, in particular, present a range of features and thus also several possible applications, with spiders being cannibalistic [23], spider silk fibers can be very cumbersome and costly to produce on a larger scale [24]. Artificial spider silk fibers based on recombinant proteins present a tantalizing option for obtaining protein-based fibers with tailored mechanical properties while avoiding the production issues of native spider silk. Thus, as recently reviewed, recombinant silk constructs based on spider silk proteins as well as on silk moth silk are currently being developed and studied for use in a range of applications [25]. However, challenges remain in the design and production process to achieve fibers with strengths as high as those that have been found for native silk fibers [26].

Various properties, such as molecular alignment, internal organization and the morphology of protein fibers, may contribute to the ultimate mechanical strength, although to what relative extent different possibly cumulative factors contribute is difficult to determine. The tensile strength of protein fibers can be determined experimentally by tensile testing, whereby a fiber is pulled until breaking while measuring its mechanical response to the applied strain creating a stress–strain curve. The strength can then be calculated from the stress–strain curve given a known fiber diameter. An estimation of the diameter can be obtained in a straightforward manner using regular light microscopy, or in more detail using electron microscopy or atomic force microscopy [27]. However, if fibers are not perfectly uniform along their entire length, a representative estimation of the average diameter can only be derived from measuring at multiple points along the length. Multiple measurements will come closer to the true average diameter, but manually measuring at multiple individual points has the evident drawback of being both a time-consuming and subjective process as well as prone to errors due to varying fiber morphology.

As is already well established for polymeric structures in general, an increase in structural alignment in protein superstructures can be linked directly to an increase in birefringence [28,29,30,31]. Well-ordered anisotropic structure sections in a sample will appear bright against a dark background when viewed with crossed polarizers. This feature has been used as a method of classification based on polarized microscopy (POM) for a variety of soft materials and structures, including liquid crystalline phases [32], films [33], gels [34], protein aggregates and spherulites [35,36,37,38]. Several studies have also previously investigated the birefringence of both natural and synthetic fibers [39,40,41,42]. Such studies have provided indications of a link between fiber tensile strength and increased birefringence [31,43,44,45]. However, the local macroscale variations observed in natural silk fibers from silk moths as well as spider silk, such as shape, thickness, twists or deformations, can cause variations in the observed optical properties in addition to reducing the general accuracy of diameter determination [46,47,48]. This is true also for artificially spun fibers that can be far from perfectly uniform throughout their entire length [49,50,51]. A calculation using the measured diameter to determine the cross-sectional area in order to calculate the engineering stress will be inherently imperfect for irregularly shaped fibers. To obtain a calculated strength that is as representative as possible for the fiber, the determination of fiber size is crucial. Single-point manual measurements of fiber diameter or birefringence are likely to yield non-representative results. In this study, we instead develop a semi-automated image-analysis procedure that avoids the time-consuming and subjective manual determination of these properties and that could be used for non-destructive quality control as well as for estimating fiber tensile strength.

## 2. Materials and Methods 

### 2.1. Protein Expression, Purification and Spinning of NT2RepCT Fibers

The expression and purification of the recombinant protein NT2RepCT was performed as described earlier [52]. After purification, NT2RepCT was concentrated to 300 mg/mL using an Amicon Ultra-15 centrifugal filter unit (Merck-Millipore, Darmstadt, Germany) equipped with an ultracel-10 membrane (10 kDa cutoff) at 4000× *g* and 4 °C. The final concentration of the protein solution (dope) was calculated with the protein-specific extinction coefficient of 18,910 M^−1^ cm^−1^ after carefully diluting the dope 500 times into 20 mM Tris (pH 8) and measuring the absorbance at 280 nm in triplicates. Then, the dope was transferred into a 1 mL syringe Luer Lock tip (BD, Franklin Lakes, NJ, USA) and connected via a 27 G blunt end steel needle (B. Braun, Melsungen, Germany) with an outer diameter of 0.40 mm to silicon tubing as detailed in Greco et al. (2020) [53]. At the end of the silicone tubing a pulled glass capillary with a tapered tip and an opening between 39 and 67 µm was inserted. The spinning dope was extruded through the glass capillary at 17 µL/min into an 80 cm long spinning bath containing 4 L of a 750 mM acetate buffer at pH 5. Fibers were collected continuously at the end of the bath with frames placed on a rotating wheel with a circumference of 35 cm as detailed in Greco et al. (2020) [53], at 50 rpm to 120 rpm (29 and 69 cm/s, respectively).

### 2.2. Native Silk Materials

*Bombyx mori* silk was prepared according to a previously established protocol [54].

The dragline silk from a Swedish bridge spider (*Larinioides sclopetarius*) was collected from live spiders. The spiders were first anesthetized with CO_2_, placed on a wax plate and immobilized. The dragline silk emerges from the anterior spinneret [23], so the silk from this spinneret was gently pulled out with a tweezer under a Zeiss Stemi 305 stereo microscope and was collected onto a rotating diapositive slide frame mounted on a motorized roller at 7.5 m/min. 

### 2.3. Light Microscopy

Two separate light microscope setups were used, referred to as setup 1 and 2.

**Figure 1:**
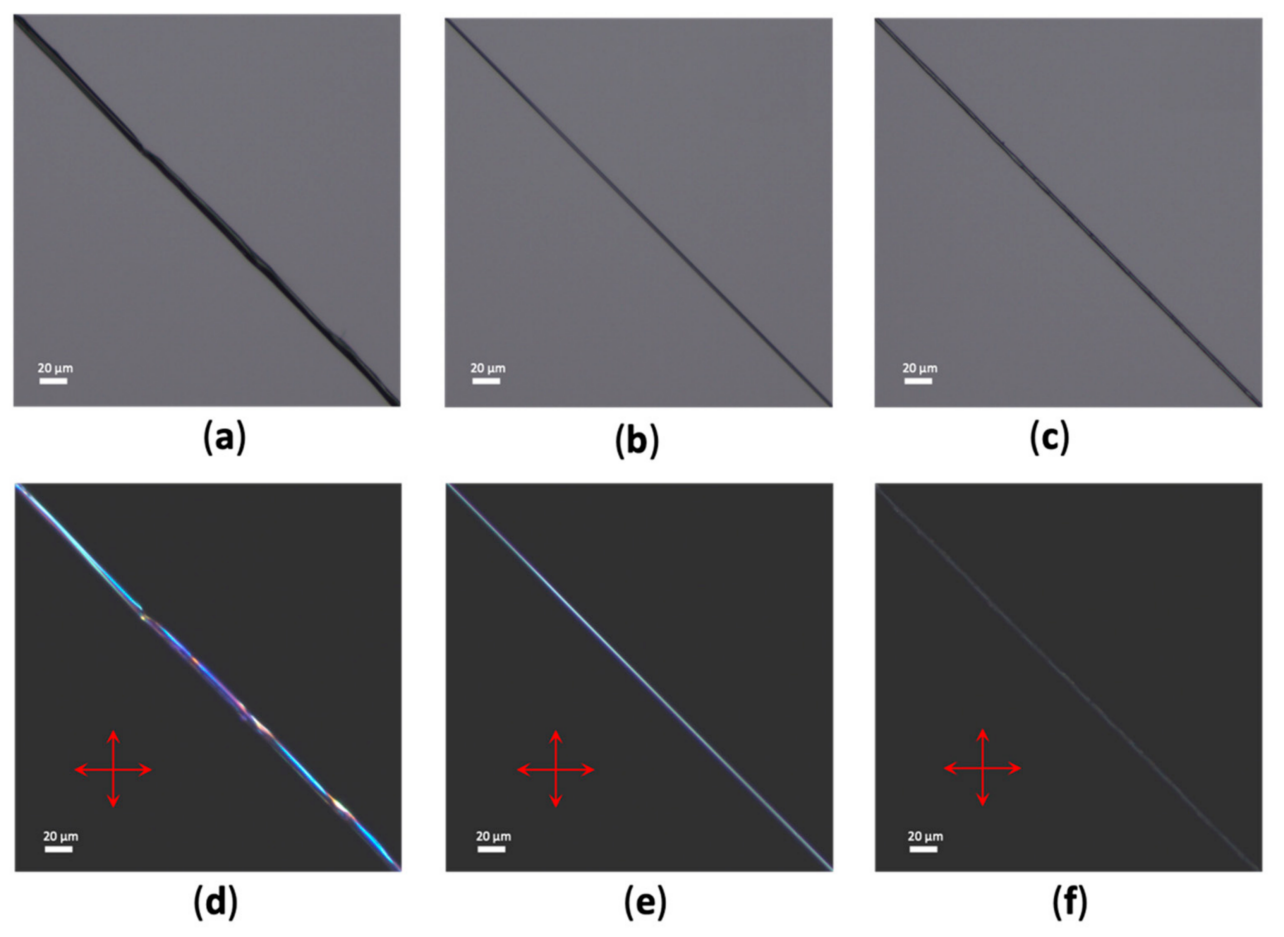
Representative light microscopy images of different fiber types were captured using the same settings. (**a**) Bright field image of a *B. mori* fiber. (**b**) Bright field image of a native dragline spider silk fiber derived from *L. sclopetarius* spider. (**c**) Bright field image of an NT2RepCT protein fiber. (**d**) POM image of a *B. mori* fiber. (**e**) POM image of a native spider dragline silk fiber derived from *L. sclopetarius*. (**f**) POM image of a NT2RepCT protein fiber. The orientation of the polarizers relative to the fiber is indicated by red double arrows.

Setup 1: Images were collected using a Nikon Eclipse Ts2R-FL inverted microscope equipped with a DFKNME33UX2495 MP camera (Bergman Labora, Stockholm, Sweden) and a CFI Plan Fluor DL-10X objective (Bergman Labora, Stockholm Sweden)). Image capture was conducted using the Nikon NIS-Elements BR software (5.30.02). For the image analysis, the exposure time was set to 2 ms for bright field images and 50 ms for polarized microscopy (POM) images. For the comparative POM images in Figure 1, the exposure time was reduced to 10 ms to avoid the overexposure of the native fibers. The brightness and contrast levels of the POM micrographs in Figure 1d–f were instead increased simultaneously as one image post capture for improved visualization using the GIMP 2.0 software [38]. Figure S1 shows the micrographs before and after this treatment. Setup 1 was used to acquire the data in Figure 1, Figure 3 and Figure 4f, Figure S1, S10 and S12 and Tables S1 and S2.

**Figure 2:**
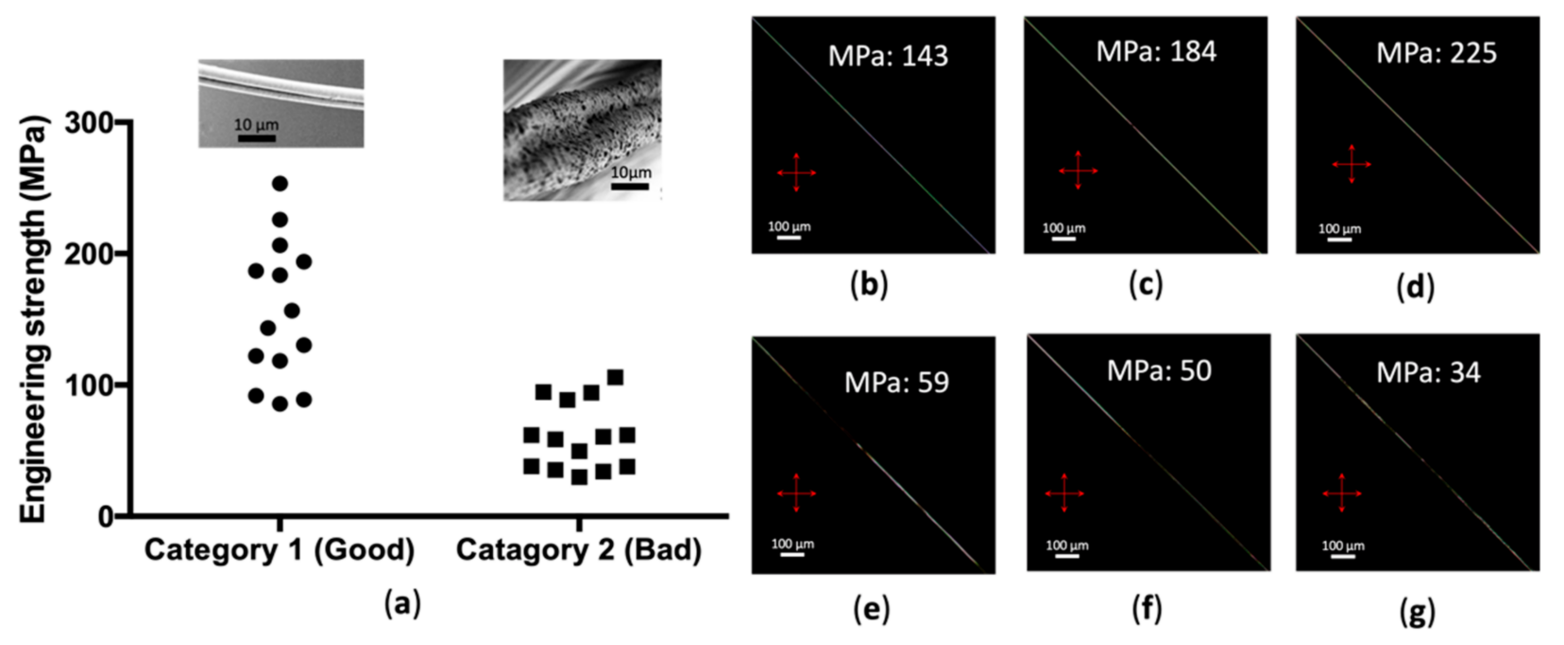
Manual sorting of fibers based on clearly visible different birefringent properties. (**a**) Plot of experimentally determined strength values for two categories of fibers. Insets: representative SEM images of fibers categorized as good and bad, respectively. (**b**–**d**) Representative POM images of fibers manually categorized as good. (**e**–**g**) Representative POM images of fibers manually categorized as bad. The orientation of the polarizers relative to the fiber is indicated by red double arrows. Insets: strength values of the fibers shown.

Setup 2: Images were acquired using a Nikon eclipse TE300 inverted microscope (Bergman Labora, Stockholm, Sweden) equipped with a DFK DFKNME33UX264 2.3 MP camera and a CFI Plan Fluor DL-10X objective (Bergman Labora, Stockholm, Sweden). Image capture was performed using the IC Measure software (2.0.0.286) from Imaging source. Setup 2 was used to acquire the POM images shown in Figure 2 and Figures S2–S7. Images acquired using setup 2 were used to generate the data in Figure S11.

Individual fibers were secured with double-sided tape to paper frames with a 10 mm gap. After mounting the fibers on paper frames, they were placed on a microscope glass slide for light microscopy imaging. For the image analysis, for both setups, the morphology was probed by bright field light microscopy and in a typical procedure, 6 sequential images were taken of the investigated fiber. Each fiber image, representing the smallest area unit over which an average value is calculated, covered approximately 1400 μm of the full fiber length. The fiber ends close to the attachment point of the paper frame were excluded to avoid interference on the image from the frame. Immediately after acquiring a bright field micrograph, a corresponding polarized microscopy image was acquired. For comparative analyses using both setups, all settings for exposure time, lamp light intensity and gain were kept the same for bright field and polarized microscopy, respectively. 

### 2.4. Image Analysis

The image analysis script was designed and written in collaboration with the BioImage Informatics Facility, SciLifeLab, Sweden and designed as a FIJI macro pipeline for the FIJI image analysis software [55]. The script as well as instructions for use can be accessed through Github (https://github.com/BIIFSweden/ProteinFiberAnalysis, accessed on 29 October 2021). For each image pair consisting of a brightfield image and a POM image counterpart of the exact same area of the fiber, the script was designed to 1 (identify the fiber part of the image using the brightfield image and determine the fiber dimensions) and 2 (analyze the birefringent properties in the form of light intensity of the corresponding fiber region of the POM image of the image pair). For the segmentation of the fiber area in the bright field images as well as for the segmentation of bright areas in the POM images, the script was designed to identify two separate threshold values to determine the border of the fiber area in the bright field images and the bright areas in the POM images, respectively. The script has an option for using Yen’s thresholding method [56] as well as an alternative fixed user defined threshold value. After using the auto threshold method to identify a threshold value separating foreground from background and manual inspection of the resulting segmented fiber areas, a threshold value (148 and 103 for setups 1 and 2, respectively) was fixed and used for the analysis of all the fiber images. Bright areas in POM images were defined by a threshold value so that all the area segments within a fiber above the threshold were defined as bright. 

For each bright field image analyzed, the script yields the identified fiber area, average fiber diameter and its corresponding average standard deviation, minimum diameter and the threshold value used. The diameter was calculated using Euclidian distance transform (EDT) [57]. For each POM image, the script yields the average intensity of the full fiber image and its corresponding standard deviation as well as the average intensity of only bright area segments. For bright area segments derived from a second segmentation process applied to the fiber region previously identified from the bright field image, the script also yields the average intensity of each identified segment as well as the mean, minimum and maximum bright area sizes. From the values directly given by the output of the script, the corresponding parameter values for all the images of a given fiber as well as additional combination parameters were put together using Microsoft Excel. Acquired images for setups 1 and 2 can be found at FigShare.

### 2.5. Statistical Analysis

Scatter plots were generated and the corresponding correlation coefficient analyses and curve fittings were performed using GraphPad Prism version 6.00 [58]. Curve fitting was performed with a model for simple linear regression after comparison with a quadratic model by a likelihood-ratio test (*p* = 0.12). With no significant benefit in using a quadratic model from a statistical point of view, the simpler model was chosen. All correlation coefficient values (Pearson) are reported as absolute values as the magnitude regardless of the sign informs on the degree of correlation.

### 2.6. Tensile Testing

Individual fibers were secured with a double-sided tape to paper frames with a 10 mm gap in preparation for pulling experiments. The mechanical properties of fibers previously characterized with light microscopy were determined using a 5943-Instron tensile tester machine and a 5 N load cell. The pulling rate was 60 mm/min (~1%/s) and the ambient temperature was 21 to 22 °C. To not affect the mechanical properties, tensile tests were exclusively performed at a relative humidity of 35% or lower [59]. This previous study saw no significant effect of water on fibers below 50% relative humidity [59]. Average fiber diameter values obtained from the image analysis procedure were used to calculate the engineering stress as the measured applied force over the cross-sectional fiber area. The engineering strain was calculated by dividing the imposed displacement for the gauge length (10 mm). In the engineering stress–strain curve, the ultimate strength is represented by the last point before fracture. From what we observed of the protein fibers in this study, as well as in previous works [52,53,59], NT2RepCT fibers have a non-circular cross-section with a longitudinal groove, as seen in Figure S8a. As depicted in Figure S8b, measuring the diameter in the face of a fiber with such a groove will result in an overestimation of the true area. Thus, the calculated stress and strength will be underestimated. Representative stress–strain curves are shown in Figure S9, with a notable absence of a so-called toe region [60].

### 2.7. Scanning Electron Microscopy (SEM)

For SEM characterization, a FE-SEM Zeiss—40 Supra was used. The metallization was performed with the Quorum Q150T sputtering machine (sputtering mode: Pt/Pd 80:20 for 5 min (Judges Scientific, Laughton, UK)). The samples were prepared according to a previously established protocol [54].

## 3. Results

Silk fibers of different origins can be difficult to tell apart by brightfield light microscopy (Figure 1a–c), but when investigated with polarized microscopy (POM) the level of birefringence is notably much higher for *B. mori* fibers (Figure 1d) and dragline silk fibers (Figure 1e) as compared to the recombinant fibers investigated in this study (Figure 1f). Silk moth and spider silk fibers typically have tensile strength values several times that of the recombinant silk fibers investigated in this paper [6,16,19,61,62]. We thus hypothesized that birefringence can be used as a convenient tool to predict the tensile strength of recombinant silk fibers.

To investigate if the birefringence of the recombinant silk fibers can serve as an indicator of tensile strength, as a first experiment, we visually sorted fibers spun from the recombinant protein NT2RepCT [63] as ‘good’ or ‘bad’ based on whether they displayed homogeneous and strong birefringence (Figure 2b–f). Subsequent tensile tests of the fibers (performed with blinded samples) revealed that the fibers sorted as bad had significantly lower engineering strength than the good fibers that showcased close to continuous high intensity when viewed with crossed polarizers (Figure 2a). The full set of POM images of the fibers in Figure 2b–g are shown in Figure S2–S7. Representative scanning electron microscopy (SEM) images of fibers selected based on the birefringence as good and bad are shown in Figure S8.

**Scheme 1:**
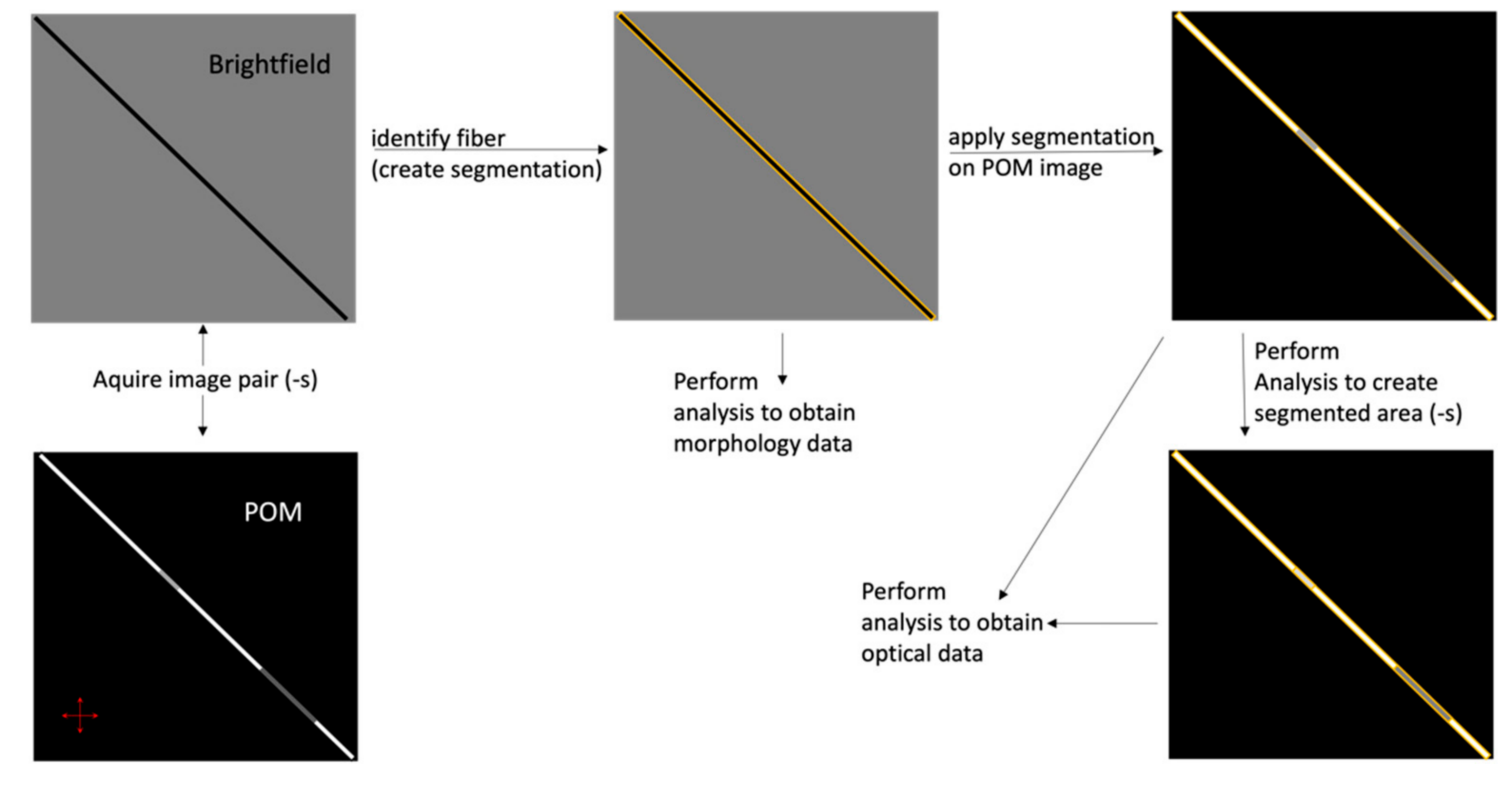
Schematic drawing of the workflow for the semi-automated image analysis process. A pair of light microscopy images of a fiber—one obtained in bright field mode and one with crossed polarizers—is acquired, followed by an automated image analysis to generate data on the fiber regarding morphological and optical characteristics. The bright field image is then utilized for determining the border of the fiber and performing a segmentation resulting in a binary mask (outlined in yellow). Multiple sequential image pairs can be acquired to cover longer fiber sections and subsequent image analysis is performed.

To investigate if we could establish a method that could be used to predict a fiber’s tensile strength utilizing birefringence, we developed a semi-automated image analysis procedure by creating a computer image analysis script where the morphology and the birefringent properties of multiple recombinant fibers were analyzed concomitantly. The process is outlined in Scheme 1. This approach allows the analysis of a long fiber section by acquiring multiple photos along a fiber’s length. The need for this was evident since the birefringence of the recombinant fibers could vary significantly along the length of the fiber (as exemplified in Figure 2e–g).

The script was designed to generate data on the morphology of the fibers by identifying the fiber in a bright field micrograph and analyzing the dimensions of the resulting fiber area. Initially, Yen’s auto thresholding method was used to identify a threshold value separating the bright background from the dark fiber [56]. Automatic thresholding worked very well for finding an approximate threshold value on a new system or with new settings. In this study, special care was taken to minimize any variations in background intensity by maintaining the same settings for the acquisition of comparative images. Furthermore, although Yen’s is a well-established algorithm for automatically finding a value separating a dark object from a bright background, or vice versa, using a fixed value for segmentation improved the script’s ability to avoid the false identification of local light shadows in a bright field image as part of the fiber. Thus, for this study, following the use of the algorithm for finding a threshold value, a threshold value was fixed and used for all further analyses. Data on the birefringent properties were generated by analyzing the light intensity within the segmented fiber area. In addition, to direct intensity related data, a further analysis was conducted by dividing the area within the fiber into bright and dark areas defined as brighter or darker than a given cut-off value. See the Methods Section for set values and a link to a detailed instruction of script use.

**Figure 3:**
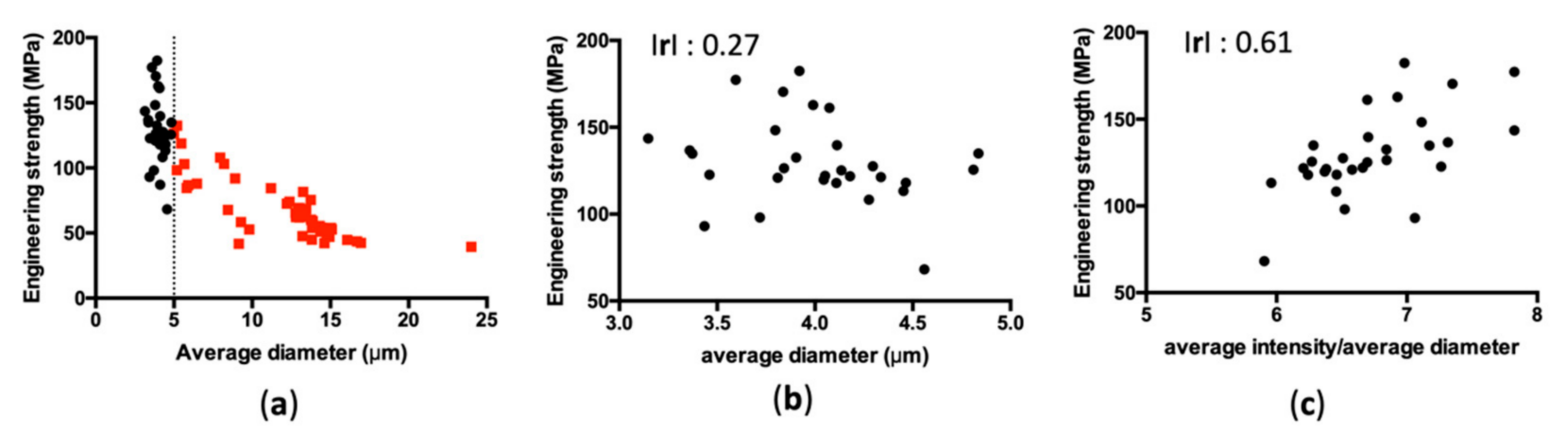
Sorting fibers based only on the size and the effect of also considering birefringence. (**a**) Engineering strength plotted against average fiber diameter. Fibers with a diameter larger than 5 µm are shown as red solid squares. (**b**) Average diameter plotted against engineering strength for fibers of a diameter < 5 µm. (**c**) Average intensity normalized to fiber diameter plotted against engineering strength for the fibers in Figure 3b. Insets: correlation coefficient values.

For the fibers investigated in this study, there was a general overall inverse correlation between size (fibers ranging in diameter from 3.1 to 24.0 µm; see Figure 3a) and strength. However, looking at fibers with a diameter of less than 5,0 µm, the correlation between diameter and engineering strength was notably low (see Figure 3b, r = 0.27). In contrast, when considering these fibers in terms of their average birefringence intensity normalized to their respective average diameter, the correlation to the engineering strength improved significantly (Figure 3c, r = 0.61).

**Figure 4:**
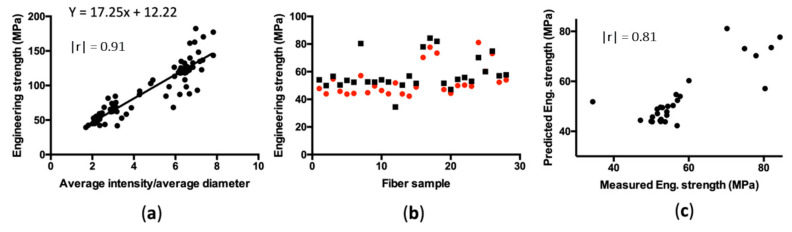
The process for the prediction of recombinant spider silk fiber tensile strength values. (**a**) Curve fitting to a scatter plot of normalized birefringence intensity plotted against measured engineering strength. (**b**) The predicted engineering strength of the individual fibers (red solid circles) based on the equation derived from Figure 4a and the corresponding measured engineering strength (black solid boxes). (**c**) Predicted engineering strength plotted against measured engineering strength. Insets: equation used for prediction and correlation coefficient values.

Based on the values of normalized intensity, a curve fitting was performed on the data in Figure 4a to generate an equation for the prediction of fiber tensile strength. A new test set of 28 recombinant fibers from a new batch of proteins were subsequently subjected to tensile testing in a blind manner. The result from the prediction compared to the corresponding measured values are shown in Figure 4b,c and Table S2. The average percentual difference between the measured and predicted values in Figure 4b was 9% and the correlation coefficient r, when plotting the predicted against experimentally determined engineering strength, was 0.81 (Figure 4c). The predicted strength based only on diameter plotted against measured strength is shown for reference in Figure S12 (r = 0.51). Figure 4 conclusively shows that the image analysis procedure we developed can be used to predict the tensile strength of recombinant spider silk fibers with a high degree of accuracy.

Looking at the full data set of recombinant fibers, a series of parameters based on the identified bright areas relative to the total fiber area were also investigated for a correlation to fiber strength (the parameters and their description are listed in Table S1). However, the correlation to engineering strength of these parameters (see Figure S10 and Table S1) were found to be inferior to the normalized intensity. To verify the robustness of the method, a separate set of recombinant fibers was investigated using a second older light microscope setup. Additionally, in this setup, a correlation between the normalized intensity and engineering strength could be observed (r = 0.70, see Figure S11).

## 4. Discussion

To the best of our knowledge, this is the first time an image-analysis-based method has been successfully developed and implemented for predicting the engineering strength of artificial silk fibers. In contrast to a previously published prediction method based on molecular simulations [64], our method gives predictive values for individual fibers spun from the same precursor protein solution and can be used as a non-disruptive quality control procedure post spinning. A less time-consuming procedure for evaluating the quality of fibers post spinning compared to tensile testing will also be useful in future attempts to optimize spinning procedures, which should be conducted to reduce the variability in fiber properties observed even when using the same spinning set-up [49,50,51,53].

As shown in Figure 1 and Figure 2, birefringence alone can be used for manual classification and rough sorting of protein fibers. However, as evident from Figure 2e–g, for birefringence to be used for an accurate prediction of a fiber’s tensile strength, a more detailed analysis considering the normalized birefringence effect along the fiber length is essential. Regarding fiber morphology, obtaining the exact cross-sectional area along a non-homogeneous fiber is a difficult task that will require further studies and experimental set-ups. However, the method developed in this paper ensures that we get a significantly better estimate of the fiber diameter, as it is measured for every pixel along the captured fiber length. This is in stark contrast to current methods where the average diameter is calculated from 3–10 randomly selected points along the fiber. Increased material size increases the probability of internal structural defects and thus promotes lower tensile strength [65]. This would explain a general decrease in strength with increasing fiber size, an overall trend also previously observed for other types of fibers [15,66,67]. However, as illustrated in Figure 3b,c, using the protein fiber size alone for estimating fiber strength was inferior to using normalized birefringence intensity. In contrast, an estimation of fiber tensile strength by also considering large-scale structural alignments in the form of birefringence improved the estimation. This supports the idea that, for the successful design and production of artificial silk fibers of high strength, the material composition and organization should be prone to well-ordered alignment, increasing the likelihood of strengthening intermolecular interactions. At first glance, for protein fibers, a higher degree of alignment might appear to require and only occur for fibers with a high crystalline content, which for silk protein fibers would mean a higher content of ß-sheets. A high content of crystalline components in the form of ß-sheets is indeed likely to be found in silk fibers with high mechanical strengths [58,68,69], a trend also recently observed for recombinant artificial silk fibers [70]. However, alignment on a large scale and the strength of fibers may not be related so much to the absolute percentage of ß-sheets [71], but rather to how they organize into larger structures [43]. The comparison of silks from silk moths with spiders silks has also shown that, although they contain a similar fraction of ß-sheet, the spider silks can have a significantly higher strength [6]. This is possibly due to differences in the internal organization that could, e.g., enable an increased number of beneficial intermolecular crosslinks [69,72]. This likely applies not only to crystalline structural components, such as ß-sheets, but also to more amorphous components of silk fibers. The varying degree of large-scale ordered alignment of such non-crystalline structures has previously been shown to have a significant impact on the mechanical properties of spider silk fibers, with a higher degree of order correlating with beneficial mechanical properties [73]. Birefringence, unlike secondary structure content analysis, could thus reveal a structural organization beneficial for a higher strength originating with (but not differentiating between) both crystalline and amorphous structural content as it is based on structural alignment rather than content. The analysis of fiber properties enabled by brightfield and polarized microscopy images stresses the significant impact that observable structural defects and ordered alignment have on the tensile strength of fibers. Image analysis of fiber micrographs alone can provide enough information to identify and sort fibers of varying strength and thus be useful as a quality control procedure, despite not, by itself, fully elucidating the complex underlying molecular interactions ultimately leading to such differences.

For a more in-depth understanding of the specifics of structure function relationship in protein fibers, other established experimental methods could in future studies act as a valuable complement to the image analysis procedure. Detailed structural analyses using nuclear magnetic resonance spectroscopy or X-ray diffraction methods have the potential to elucidate the relationship between structure and function in fibers more clearly, although they by themselves have limited value as high throughput quality control techniques. Secondary structure content analysis using, e.g., Fourier transform infrared spectroscopy (FTIR) or Raman spectroscopy could add information on a possible connection between secondary structures and alignment. Although challenging techniques in practice for the evaluation of individual fibers, if coupled with a better concomitant understanding of large-scale organization, they could then help to better explain the mechanical properties of silk-like fibers [74].

For the determination of the tensile strength of fibers, a general challenge is that local defects can have an impact on the tensile strength of a fiber as a whole [62]. Any approach to determine the fiber tensile strength, either experimental tensile testing or predictive, that does not account for a large section of a fiber’s length is likely to miss this effect [15,75]. Unfortunately, conventional tensile testing by design destroys the sample in the process. An alternative method for measuring strain and investigating the mechanical properties based on image analysis that could be employed is the digital image correlation (DIC) method that has previously shown promise for use with bio-based structures [59]. However, as with conventional tensile testing, this method would require a sample to be isolated and subjected to structural alterations, thus preventing an analysis on a pristine spun fiber and the development of an automated quality control procedure. By instead using the image analysis script developed in this paper and a common light microscope equipped with polarizers, predictive values for fiber tensile strength could be achieved in a facile and precise manner on pristine fibers. As showcased in Figure 4, simply considering the average intensity of a fiber normalized to its average diameter can enable a highly potent estimation of a fiber’s tensile strength. Subsequently, the non-destructive feature of the methodology developed in this study enables intact fibers to be investigated further by other techniques. This is a proof of concept for a simple and inexpensive way to estimate protein fiber tensile strength that could also act as a commentary method of analysis useful for future fundamental research into the structure–function relationship of fibers. Lastly, our image analysis method could be developed into a fully automated high-throughput quality control procedure for the commercial production of protein fibers and possibly also different types of bio-based fibers.

## Data Availability

Raw data in the form of micrographs are available through FigShare (10.6084/m9.figshare.c.5736620).

## Supplementary Materials

The following supporting information can be downloaded at https://www.mdpi.com/1996-1944/15/3/708/s1. Figure S1: Images captured with setup 2 before and after increasing brightness and contrast for improved visualization [76]; Figure S2: Sequential non-overlapping gray scale POM images of the fiber from Figure 2b; Figure S3: Sequential non-overlapping gray scale POM images of the fiber from Figure 2c; Figure S4: Sequential non-overlapping gray scale POM images of the fiber from Figure 2d; Figure S5: Sequential non-overlapping gray scale POM images of the fiber from Figure 2e; Figure S6: Sequential non-overlapping gray scale POM images of the fiber from Figure 2f; Figure S7: Sequential non-overlapping gray scale POM images of the fiber from Figure 2g; Figure S8a: Representative SEM images of fibers sorted as good and bad; Figure S8b: Schematic drawing of the measuring of a fiber cross-section diameter with a light microscope; Figure S9: Representative engineering stress–strain curves; Figure S10: Scatter plot of the bright area parameter that yielded the highest correlation when plotted against the measured engineering strength; Figure S11: Average intensity over average diameter plotted against engineering strength for an extra set of fibers using setup 2; Figure S12: The predicted strength based only on the diameter plotted against the measured strength; Table S1: Correlation coefficient values for bright area parameters for the fibers underlying Figure 4a; Table S2: Predicted values and the corresponding measured engineering tensile strength values of Figure 4b,c.

## References

[1] Fang G., Sapru S., Behera S., Yao J., Shao Z., Kundu S.C., Chen X. (2016). Exploration of the Tight Structural-Mechanical Relationship in Mulberry and Non-Mulberry Silkworm Silks. J. Mater. Chem. B.

[2] Swanson B.O., Blackledge T.A., Beltrán J., Hayashi C.Y. (2006). Variation in the Material Properties of Spider Dragline Silk across Species. Appl. Phys. A Mater. Sci. Process.

[3] Fu C., Porter D., Chen X., Vollrath F., Shao Z. (2011). Understanding the Mechanical Properties of Antheraea Pernyi Silka-From Primary Structure to Condensed Structure of the Protein. Adv. Funct. Mater..

[4] Gosline J.M., Guerette P.A., Ortlepp C.S., Savage K.N. (1999). The Mechanical Design of Spider Silks: From Fibroin Sequence to Mechanical Function. J. Exp. Biol..

[5] Fraternali F., Stehling N., Amendola A., Anrango B.A.T., Holland C., Rodenburg C. (2020). Tensegrity Modelling and the High Toughness of Spider Dragline Silk. Nanomaterials.

[6] Guo C., Zhang J., Jordan J.S., Wang X., Henning R.W., Yarger J.L. (2018). Structural Comparison of Various Silkworm Silks: An Insight into the Structure-Property Relationship. Biomacromolecules.

[7] Cranford S.W., Tarakanova A., Pugno N.M., Buehler M.J. (2012). Nonlinear Material Behaviour of Spider Silk Yields Robust Webs. Nature.

[8] Keten S., Xu Z., Ihle B., Buehler M.J. (2010). Nanoconfinement Controls Stiffness, Strength and Mechanical Toughness of Β-Sheet Crystals in Silk. Nat. Mater..

[9] Nova A., Keten S., Pugno N.M., Redaelli A., Buehler M.J. (2010). Molecular and Nanostructural Mechanisms of Deformation, Strength and Toughness of Spider Silk Fibrils. Nano Lett..

[10] Du N., Xiang Y.L., Narayanan J., Li L., Lim M.L.M., Li D. (2006). Design of Superior Spider Silk: From Nanostructure to Mechanical Properties. Biophys. J..

[11] Liu R., Deng Q., Yang Z., Yang D., Han M.Y., Liu X.Y. (2016). “Nano-Fishnet” Structure Making Silk Fibers Tougher. Adv. Funct. Mater..

[12] Blackledge T.A., Pérez-Rigueiro J., Plaza G.R., Perea B., Navarro A., Guinea G.V., Elices M. (2012). Sequential Origin in the High Performance Properties of Orb Spider Dragline Silk. Sci. Rep..

[13] Swanson B.O., Anderson S.P., DiGiovine C., Ross R.N., Dorsey J.P. (2009). The Evolution of Complex Biomaterial Performance: The Case of Spider Silk. Integr. Comp. Biol..

[14] Greco G., Wolff J.O., Pugno N.M. (2020). Strong and Tough Silk for Resilient Attachment Discs: The Mechanical Properties of Piriform Silk in the Spider Cupiennius Salei (Keyserling, 1877). Front. Mater..

[15] Greco G., Pugno N.M. (2020). Mechanical Properties and Weibull Scaling Laws of Unknown Spider Silks. Molecules.

[16] Omenetto F.G., Kaplan D.L. (2010). New Opportunities for an Ancient Material. Science.

[17] Kluge J.A., Rabotyagova O., Leisk G.G., Kaplan D.L. (2008). Spider Silks and Their Applications. Trends Biotechnol..

[18] Allmeling C., Jokuszies A., Reimers K., Kall S., Choi C.Y., Brandes G., Kasper C., Scheper T., Guggenheim M., Vogt P.M. (2008). Spider Silk Fibres in Artificial Nerve Constructs Promote Peripheral Nerve Regeneration. Cell Prolif..

[19] Altman G.H., Diaz F., Jakuba C., Calabro T., Horan R.L., Chen J., Lu H., Richmond J., Kaplan D.L. (2003). Silk-Based Biomaterials. Biomaterials.

[20] Chouhan D., Mandal B.B. (2020). Silk Biomaterials in Wound Healing and Skin Regeneration Therapeutics: From Bench to Bedside. Acta Biomater..

[21] Kim S., Mitropoulos A.N., Spitzberg J.D., Tao H., Kaplan D.L., Omenetto F.G. (2012). Silk Inverse Opals. Nat. Photonics.

[22] Omenetto F.G., Kaplan D.L. (2008). A New Route for Silk. Nat. Photonics.

[23] Foelix R.F. Biology of Spider.

[24] Salehi S., Scheibel T. (2018). Biomimetic Spider Silk Fibres: From Vision to Reality. Biochemist.

[25] Saric M., Scheibel T. (2019). Engineering of Silk Proteins for Materials Applications. Curr. Opin. Biotechnol..

[26] Koeppel A., Holland C. (2017). Progress and Trends in Artificial Silk Spinning: A Systematic Review. ACS Biomater. Sci. Eng..

[27] Menezes G.M., Teulé F., Lewis R.V., Silva L.P., Rech E.L. (2013). Nanoscale Investigations of Synthetic Spider Silk Fibers Modified by Physical and Chemical Processes. Polym. J..

[28] Ward I.M. (1997). Structure and Properties of Oriented Polymers.

[29] Houck M.M., Siegel J.A. (2015). Fundamentals of Forensic Science.

[30] Jin H.J., Kaplan D.L. (2003). Mechanism of Silk Processing in Insects and Spiders. Nature.

[31] Lazaris A., Arcidiacono S., Huang Y., Zhou J.F., Duguay F., Chretien N., Welsh E.A., Soares J.W., Karatzas C.N. (2002). Spider Silk Fibers Spun from Soluble Recombinant Silk Produced in Mammalian Cells. Science.

[32] Cannon D., Donald A.M. (2013). Control of Liquid Crystallinity of Amyloid-Forming Systems. Soft Matter.

[33] Wang L., Bäcklund F.G., Yuan Y., Nagamani S., Hanczyc P., Sznitko L., Solin N. (2021). Air-Water Interface Assembly of Protein Nanofibrils Promoted by Hydrophobic Additives. ACS Sustain. Chem. Eng..

[34] Shu T., Zheng K., Zhang Z., Ren J., Wang Z., Pei Y., Yeo J., Gu G.X., Ling S. (2021). Birefringent Silk Fibroin Hydrogel Constructed via Binary Solvent-Exchange-Induced Self-Assembly. Biomacromolecules.

[35] Bäcklund F.G., Elfwing A., Musumeci C., Ajjan F., Babenko V., Dzwolak W., Solin N., Inganäs O. (2017). Conducting Microhelices from Self-Assembly of Protein Fibrils. Soft Matter.

[36] Shtukenberg A.G., Punin Y.O., Gunn E., Kahr B. (2012). Spherulites. Chem. Rev..

[37] Bäcklund F.G., Solin N. (2015). Tuning the Aqueous Self-Assembly Process of Insulin by a Hydrophobic Additive. RSC Adv..

[38] Bäcklund F.G., Pallbo J., Solin N. (2016). Controlling Amyloid Fibril Formation by Partial Stirring. Biopolymers.

[39] Sipe J.D., Cohen A.S. (2000). Review: History of the Amyloid Fibril. J. Struct. Biol..

[40] Sokkar T.Z.N., El-Bakary M.A. (2001). The Refractive Index Profile of Highly-Oriented Fibres. J. Phys. D Appl. Phys..

[41] Carmichael S., Barghout J.Y.J., Viney C. (1999). The Effect of Post-Spin Drawing on Spider Silk Microstructure: A Birefringence Model. Int. J. Biol. Macromol..

[42] Carmichael S., Viney C. (1999). Molecular Order in Spider Major Ampullate Silk (Dragline): Effects of Spinning Rate and Post-Spin Drawing. J. Appl. Polym. Sci..

[43] Munro T., Putzeys T., Copeland C.G., Xing C., Lewis R.V., Ban H., Glorieux C., Wubbenhorst M. (2017). Investigation of Synthetic Spider Silk Crystallinity and Alignment via Electrothermal, Pyroelectric, Literature XRD, and Tensile Techniques. Macromol. Mater. Eng..

[44] Peng Q., Shao H., Hu X., Zhang Y. (2015). Role of Humidity on the Structures and Properties of Regenerated Silk Fibers. Prog. Nat. Sci. Mater. Int..

[45] Work R.W. (1977). Dimensions, Birefringences, and Force-Elongation Behavior of Major and Minor Ampullate Silk Fibers from Orb-Web-Spinning Spiders—The Effects of Wetting on These Properties. Text. Res. J..

[46] Augsten K., Mühlig P., Herrmann C. (2000). Glycoproteins and Skin-Core Structure in Nephila Clavipes Spider Silk Observed by Light and Electron Microscopy. Scanning.

[47] Poza P., Pérez-Rigueiro J., Elices M., LLorca J. (2002). Fractographic Analysis of Silkworm and Spider Silk. Eng. Fract. Mech..

[48] Kujala S., Mannila A., Karvonen L., Kieu K., Sun Z. (2016). Natural Silk as a Photonics Component: A Study on Its Light Guiding and Nonlinear Optical Properties. Sci. Rep..

[49] Bowen C.H., Dai B., Sargent C.J., Bai W., Ladiwala P., Feng H., Huang W., Kaplan D.L., Galazka J.M., Zhang F. (2018). Recombinant Spidroins Fully Replicate Primary Mechanical Properties of Natural Spider Silk. Biomacromolecules.

[50] Liu W., Huang C., Jin X. (2015). Electrospinning of Grooved Polystyrene Fibers: Effect of Solvent Systems. Nanoscale Res. Lett..

[51] Dou Y., Wang Z.P., He W., Jia T., Liu Z., Sun P., Wen K., Gao E., Zhou X., Hu X. (2019). Artificial Spider Silk from Ion-Doped and Twisted Core-Sheath Hydrogel Fibres. Nat. Commun..

[52] Schmuck B., Greco G., Barth A., Pugno N.M., Johansson J., Rising A. (2021). High-Yield Production of a Super-Soluble Miniature Spidroin for Biomimetic High-Performance Materials. Mater. Today.

[53] Greco G., Francis J., Arndt T., Schmuck B., Bäcklund F.G., Barth A., Johansson J., Pugno N.M., Rising A. (2020). Properties of Biomimetic Artificial Spider Silk Fibers Tuned by PostSpin Bath Incubation. Molecules.

[54] Bucciarelli A., Greco G., Corridori I., Pugno N.M., Motta A. (2021). A Design of Experiment Rational Optimization of the Degumming Process and Its Impact on the Silk Fibroin Properties. ACS Biomater. Sci. Eng..

[55] Schindelin J., Arganda-Carreras I., Frise E., Kaynig V., Longair M., Pietzsch T., Preibisch S., Rueden C., Saalfeld S., Schmid B. (2012). Fiji: An Open-Source Platform for Biological-Image Analysis. Nat. Methods.

[56] Yen J.-C., Chang F.-J., Chang S. (1995). A New Criterion for Automatic Multilevel Thresholding. IEEE Trans. Image Process..

[57] Leymarie F., Levine M.D. (1992). Fast Raster Scan Distance Propagation on the Discrete Rectangular Lattice. CVGIP Image Underst..

[58] Dong Z., Lewis R.V., Middaugh C.R. (1991). Molecular Mechanism of Spider Silk Elasticity. Arch. Biochem. Biophys..

[59] Greco G., Arndt T., Schmuck B., Francis J., Bäcklund F.G., Shilkova O., Barth A., Gonska N., Seisenbaeva G., Kessler V. (2021). Tyrosine Residues Mediate Supercontraction in Biomimetic Spider Silk. Commun. Mater..

[60] Ha N.S., Jin T.L., Goo N.S., Park H.C. (2011). Anisotropy and Non-Homogeneity of an Allomyrina Dichotoma Beetle Hind Wing Membrane. Bioinspiration Biomim..

[61] Knight D.P., Vollrath F. (2001). Liquid Crystalline Spinning of Spider Silk. Nature.

[62] Cheng Y., Koh L.D., Li D., Ji B., Han M.Y., Zhang Y.W. (2014). On the Strength of β-Sheet Crystallites of Bombyx Mori Silk Fibroin. J. R. Soc. Interface.

[63] Andersson M., Jia Q., Abella A., Lee X.-Y., Landreh M., Purhonen P., Hebert H., Tenje M., Robinson C.V., Meng Q. (2017). Biomimetic Spinning of Artificial Spider Silk from a Chimeric Minispidroin. Nat. Chem. Biol..

[64] Rim N.G., Roberts E.G., Ebrahimi D., Dinjaski N., Jacobsen M.M., Martín-Moldes Z., Buehler M.J., Kaplan D.L., Wong J.Y. (2017). Predicting Silk Fiber Mechanical Properties through Multiscale Simulation and Protein Design. ACS Biomater. Sci. Eng..

[65] Weibull W. (1939). A Statistical Theory of the Strength of Materials. Handlingar 151: Ingeniörs Vetenskaps Akademien.

[66] Phoenix S.L. (1978). Stochastic Strength and Fatigue of Fiber Bundles. Int. J. Fract..

[67] Yang W., Yu Y., Ritchie R.O., Meyers M.A. (2020). On the Strength of Hair across Species. Matter.

[68] Hayashi C.Y., Shipley N.H., Lewis R.V. (1999). Hypotheses That Correlate the Sequence, Structure, and Mechanical Properties of Spider Silk Proteins. Int. J. Biol. Macromol..

[69] Rathore O., Sogah D.Y. (2001). Self-Assembly of β-Sheets into Nanostructures by Poly(Alanine) Segments Incorporated in Multiblock Copolymers Inspired by Spider Silk. J. Am. Chem. Soc..

[70] Gonska N., López P.A., Lozano-Picazo P., Thorpe M., Guinea G.V., Johansson J., Barth A., Pérez-Rigueiro J., Rising A. (2020). Structure-Function Relationship of Artificial Spider Silk Fibers Produced by Straining Flow Spinning. Biomacromolecules.

[71] Htut K.Z., Alicea-Serrano A.M., Singla S., Agnarsson I., Garb J.E., Kuntner M., Gregorič M., Haney R.A., Marhabaie M., Blackledge T.A. (2021). Correlation between Protein Secondary Structure and Mechanical Performance for the Ultra-Tough Dragline Silk of Darwin’s Bark Spider. J. R. Soc. Interface.

[72] Dicko C., Knight D., Kenney J.M., Vollrath F. (2004). Secondary Structures and Conformational Changes in Flagelliform, Cylindrical, Major, and Minor Ampullate Silk Proteins. Temperature and Concentration Effects. Biomacromolecules.

[73] Sampath S., Isdebski T., Jenkins J.E., Ayon J.V., Henning R.W., Orgel J.P.R.O., Antipoa O., Yarger J.L. (2012). X-ray Diffraction Study of Nanocrystalline and Amorphous Structure within Major and Minor Ampullate Dragline Spider Silks. Soft Matter.

[74] Lefèvre T., Rousseau M.E., Pézolet M. (2007). Protein Secondary Structure and Orientation in Silk as Revealed by Raman Spectromicroscopy. Biophys. J..

[75] Carpinteri A., Pugnø N. (2005). Are Scaling Laws on Strength of Solids Related to Mechanics or to Geometry?. Nat. Mater..

[76] The GIMP Development Team (2019). GIMP.

