# Supplementary material for: An Image-Analysis-Based Method for the Prediction of Recombinant Protein Fiber Tensile Strength
Source: Materials (Basel). 2022 Jan 18;15(3):708. doi: 10.3390/ma15030708 (PMC8915176; doi:10.3390/ma15030708)
Supplement: Supplementary file 1 [file materials-15-00708-s001.zip › materials-1519968-supplementary.pdf]

Supplementary material for

An Image Analysis-Based Method for Prediction of Recombinant Protein Fiber Tensile Strength

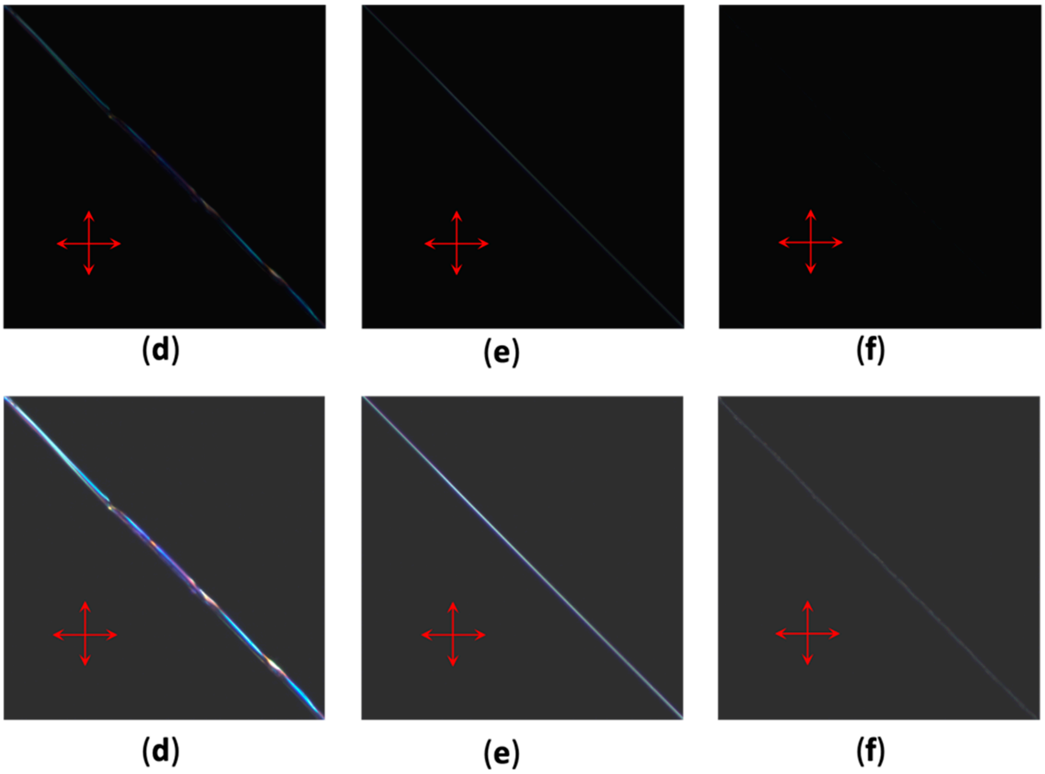

Figure S1. Images captured before and after increasing brightness and contrast for improved visualization. The three micrographs (d, e and f) were first saved as a single image after which the operation was performed on the image using the GIMP 2.0 software [76].

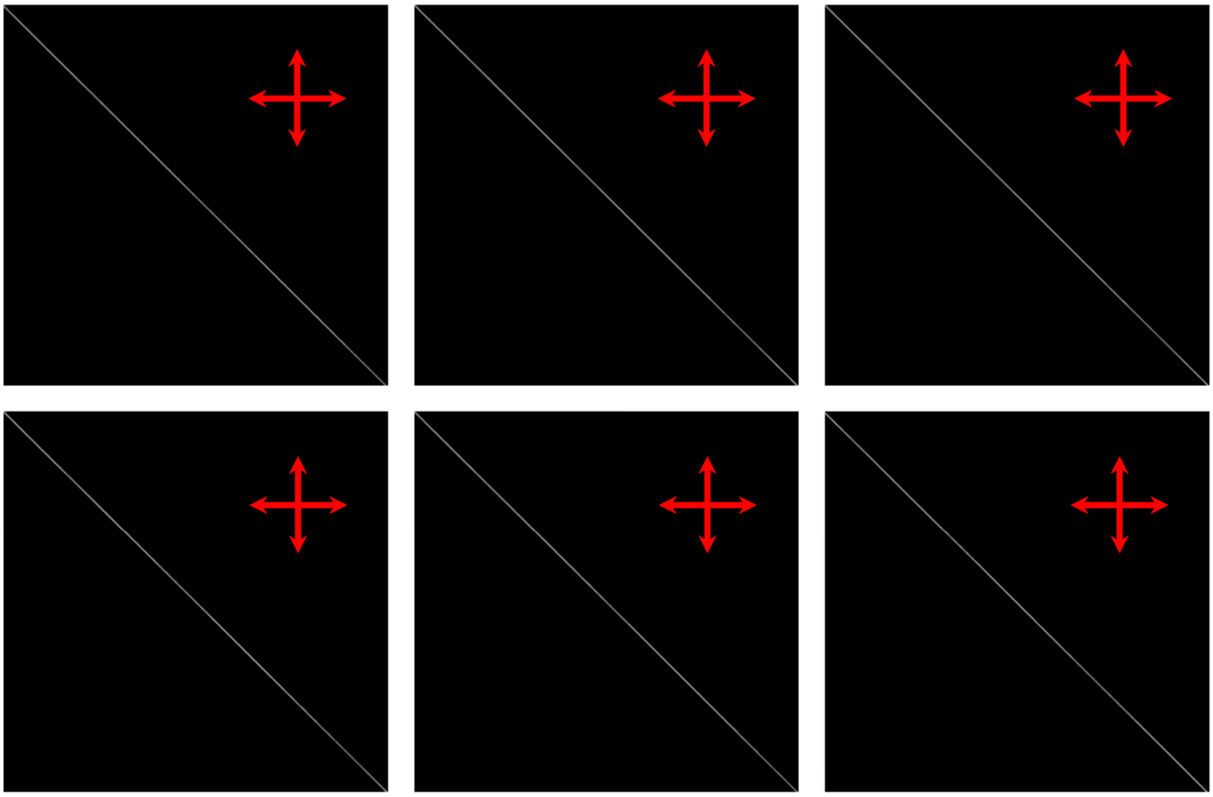

Figure S2. Sequential non overlapping gray scale POM images of the fiber from figure 2 b. Direction of polarizers indicated by red double arrows. Images captured using Setup 1 at 10 X magnification.

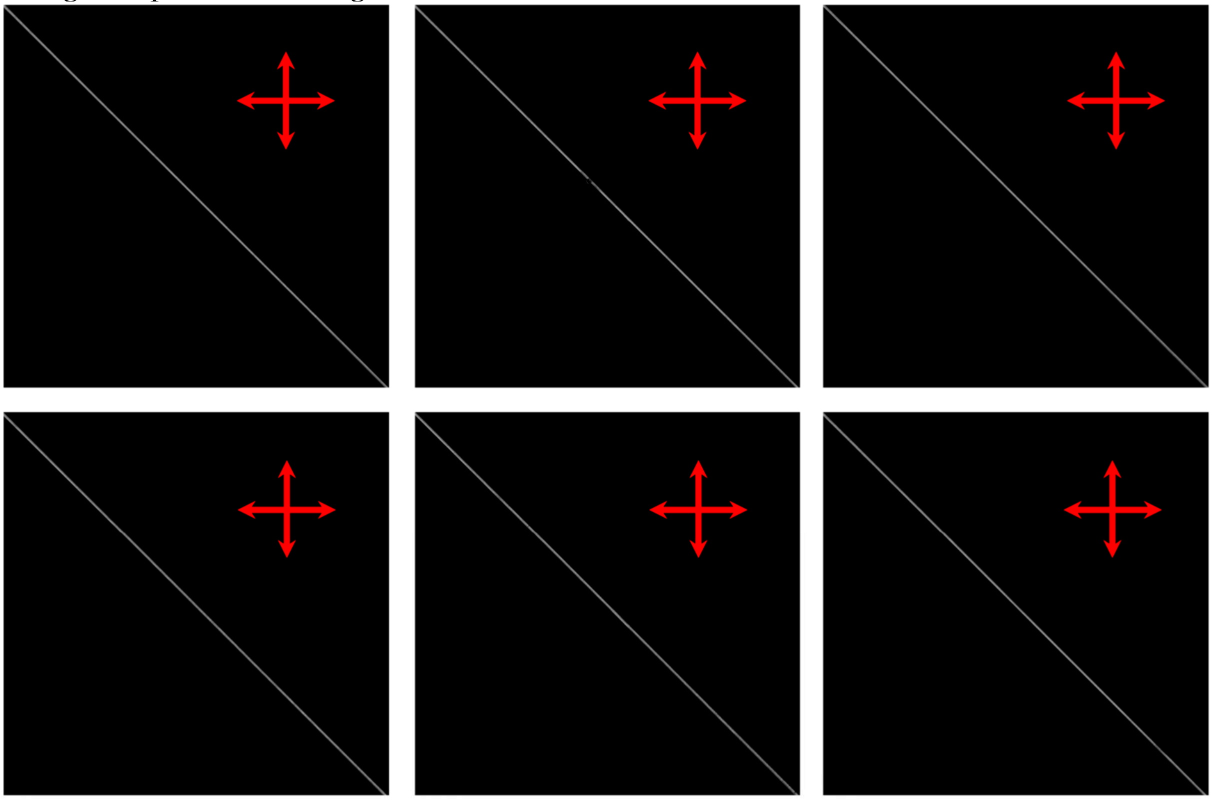

Figure S3. Sequential non overlapping gray scale POM images of the fiber from figure 2 c. Direction of polarizers indicated by red double arrows. Images captured using Setup 1 at 10 X magnification.

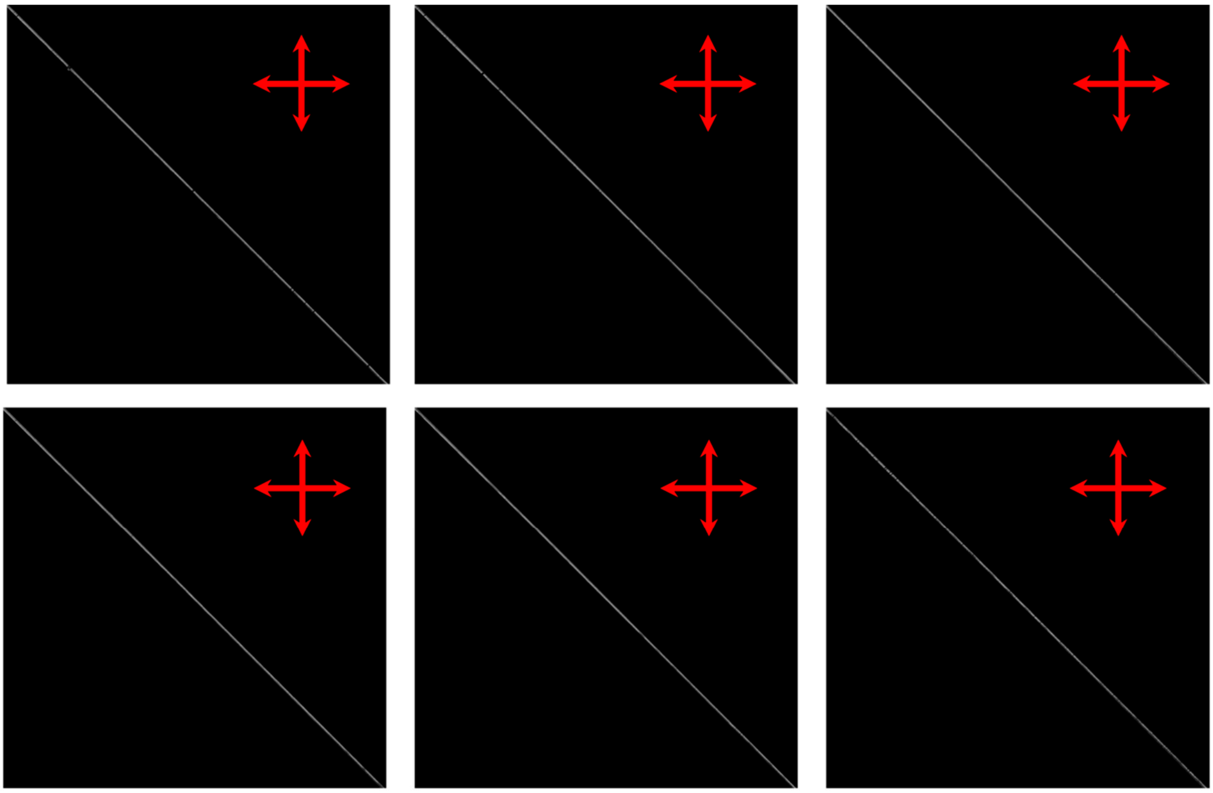

Figure S4. Sequential non overlapping gray scale POM images of the fiber from figure 2 d. Direction of polarizers indicated by red double arrows. Images captured using Setup 1 at 10 X magnification.

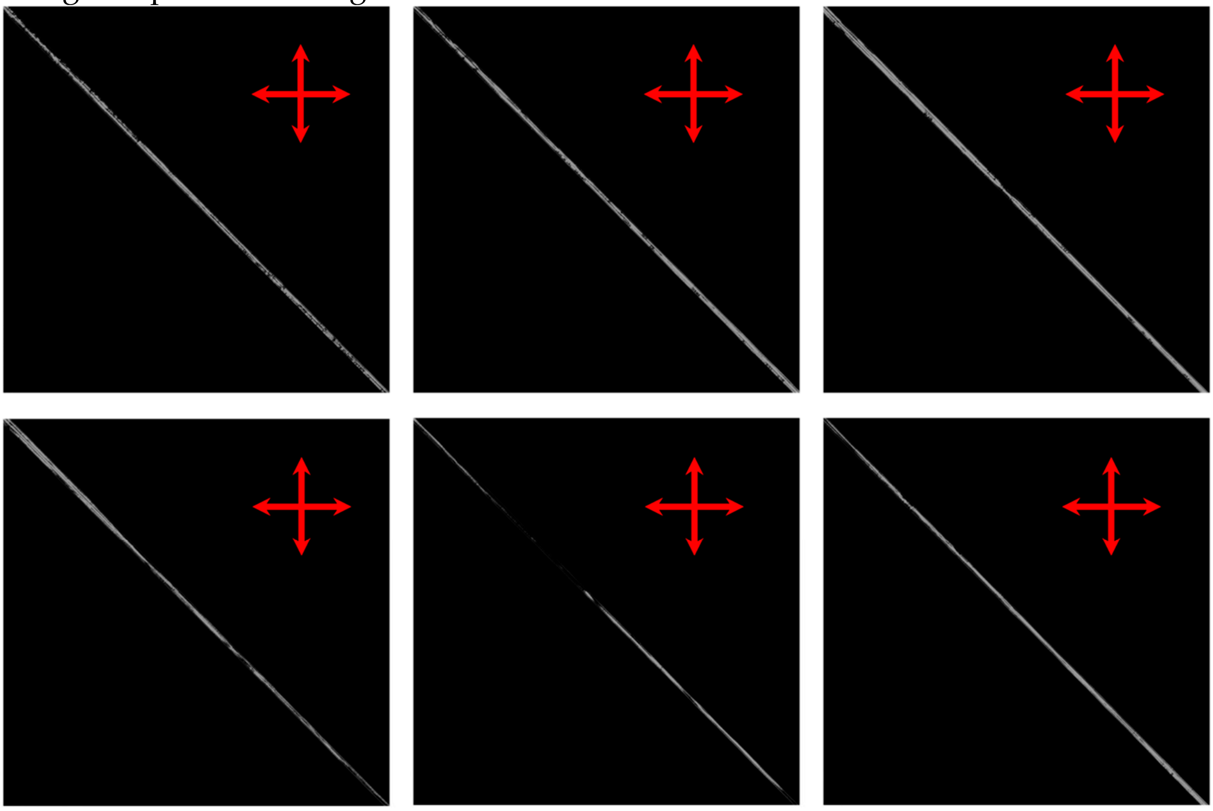

Figure S5. Sequential non overlapping gray scale POM images of the fiber from figure 2 e. Direction of polarizers indicated by red double arrows. Images captured using Setup 1 at 10 X magnification.

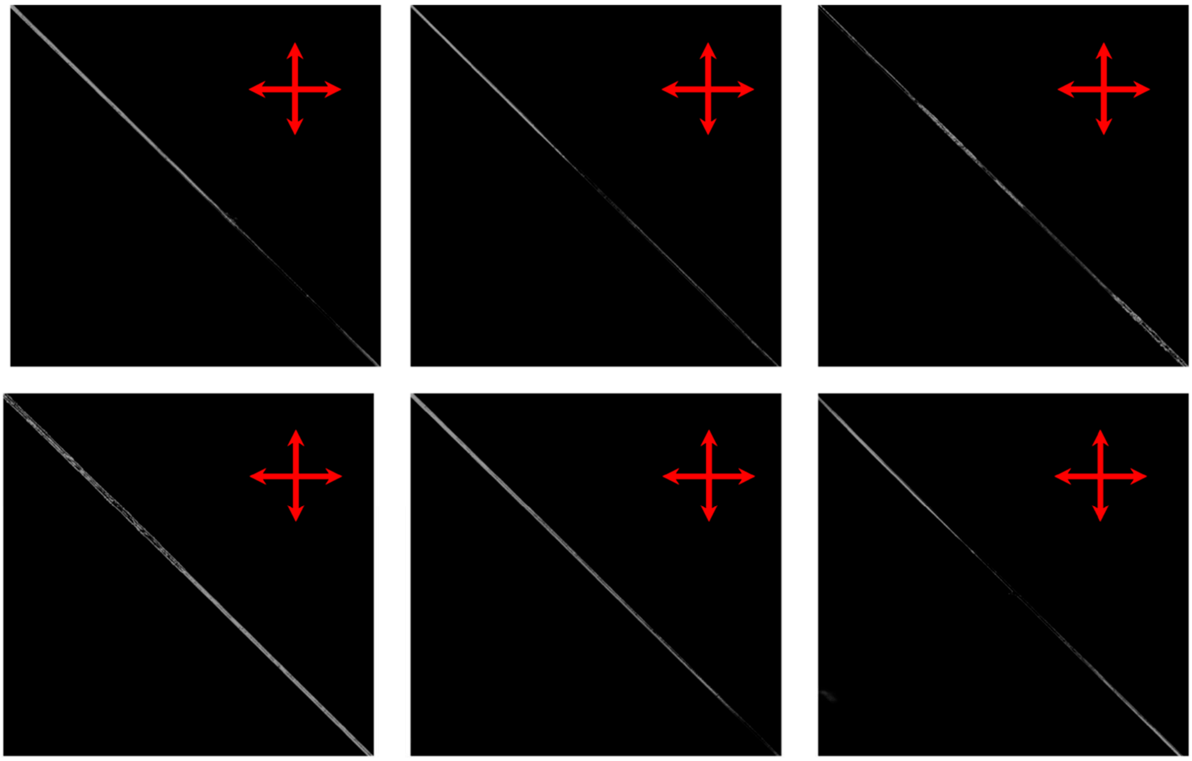

Figure S6. Sequential non overlapping gray scale POM images of the fiber from figure 2 f. Direction of polarizers indicated by red double arrows. Images captured using Setup 1 at 10 X magnification.

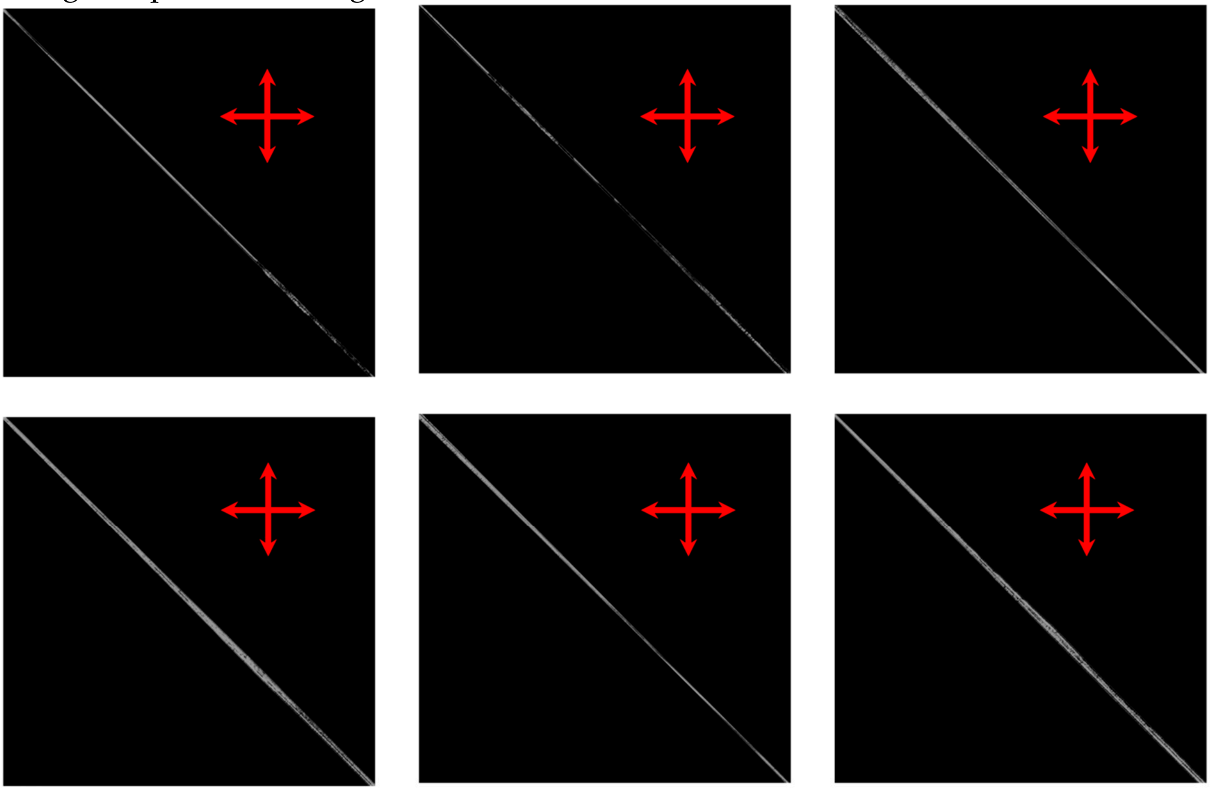

Figure S7. Sequential non overlapping gray scale POM images of the fiber from figure 2 g. Direction of polarizers indicated by red double arrows. Images captured using Setup 1 at 10 X magnification.

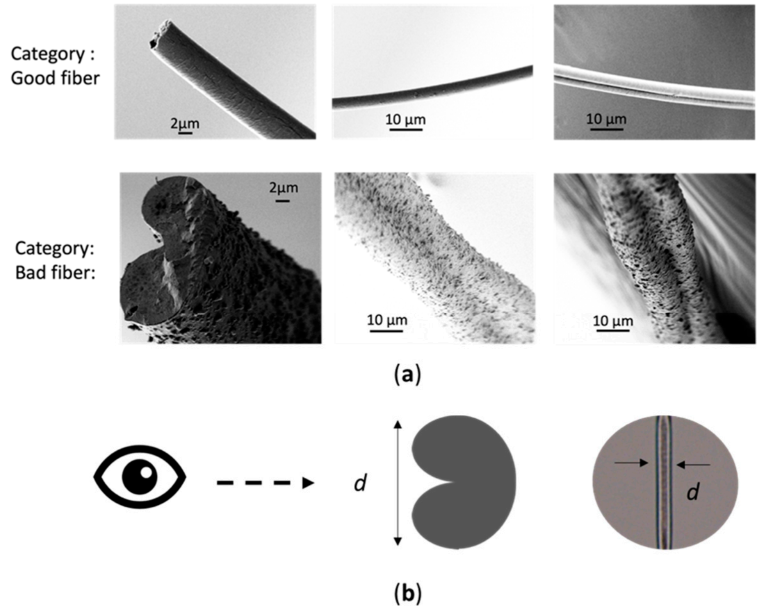

Figure S8. Representative SEM images and schematic drawing of NT2repCt fibers diameter measurement. (**a**) Representative SEM images of fibers sorted as good and bad. (**b**) Schematic drawing of measuring a fiber cross section maximum diameter with a light microscope.

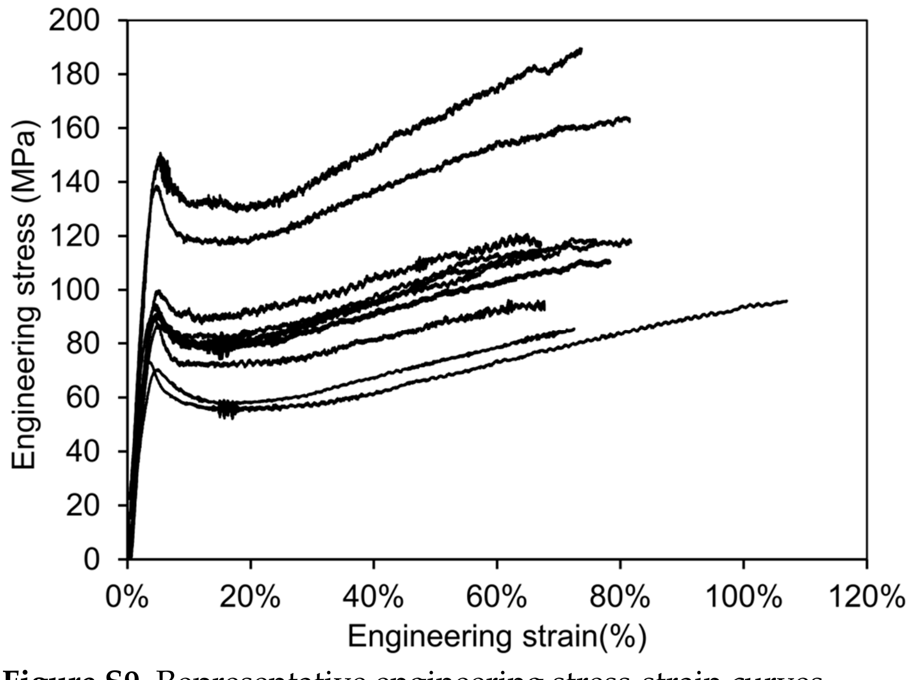

Figure S9. Representative engineering stress-strain curves.

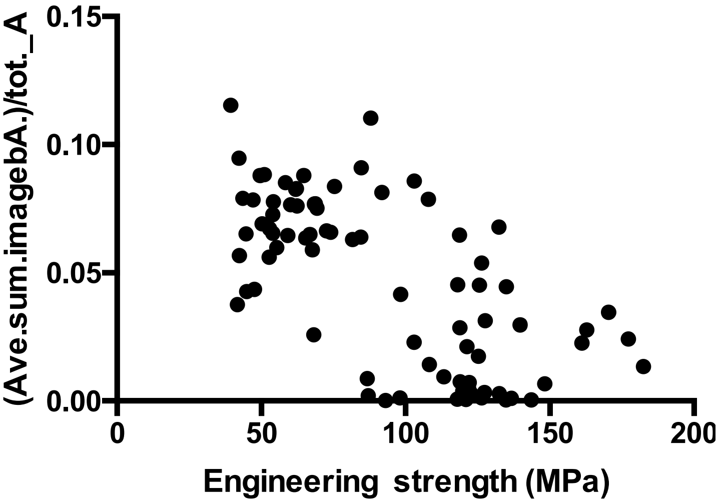

Figure S10. Scatter plot of the bright area parameter that yielded the highest correlation when plotted against measured engineering strength. (|r| = 0,66)

Table S1. Absolute values of the Pearson correlation coefficient r for bright area parameters for the fibers underlying figure 4a

Parameter	Correlation coefficient |r|
ave._bA/tot._A	0.61
Gmax._bA/tot._A	0.52
(Ave.max.bA)/tot._A	0.47
(Ave.sum.imagebA.)/tot._A	0.66
tot.bA/tot.A	0.61

ave._bA/tot._A: the average bright area identified from POM fiber images relative to the total fiber area identified from brightfield images
Gmax._bA/tot._A: the global maximum bright area identified from POM fiber images relative to the total fiber area identified from brightfield images
(Ave.max.bA)/tot._A: = the average of (the maximum bright area of each individual POM fiber image) relative to the total fiber area identified from brightfield images
(Ave.sum.imagebA.)/tot._A: average (sum of total bright area of each individual POM fiber image) relative to the total fiber area identified from brightfield images
tot.bA/tot.A: the total bright area identified from POM fiber images relative to the total fiber area identified from brightfield images

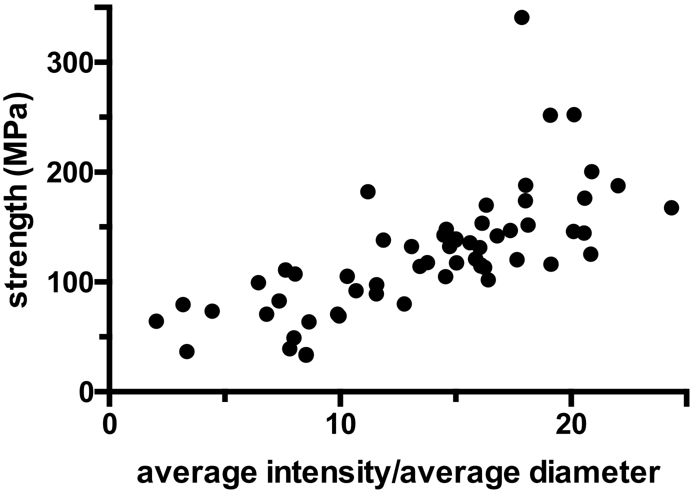

Figure S11. Average intensity over average diameter plotted against engineering strength for an extra set of fibers using setup 2. Pearson correlation coefficient r = 0.70

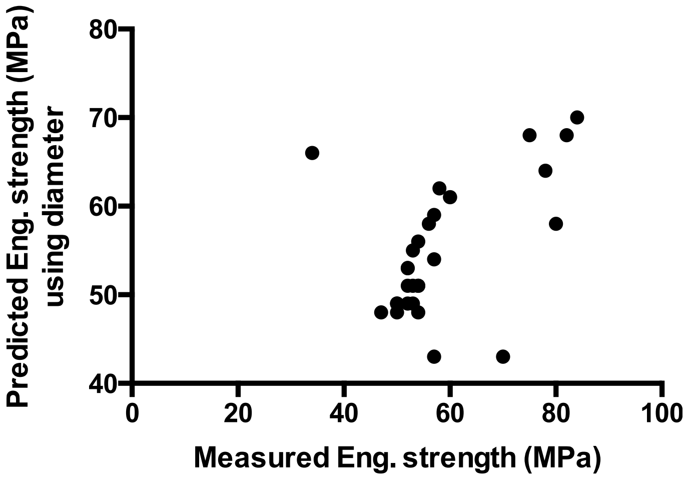

Figure S12. The predicted strength based only on diameter plotted against measured strength. Pearson correlation coefficient r = 0.50

Table S2. Predicted values and the corresponding measured engineering tensile strength values of figure 4b-c.

Measured Eng. strength (MPa)	Predicted Eng. strength (MPa)
54.14	47.80084
49.97	43.98664
56.63	54.70787
50.34	45.76067
53.74	43.80034
52.38	44.26527
80.34	57.09075
52.63	44.79338
52.47	49.63588
54.14	46.415
52.54	43.92113
34.42	51.85962
50.27	43.83798
56.91	42.25412
51.58	48.90983
77.93	70.27099
84.31	77.67467
81.98	73.49686
51.64	47.08956
47.14	44.39293
54.44	49.99828
55.82	50.32406
53	49.48837
70.21	81.11977
60.06	60.26598
74.86	73.12772
57.01	52.40215
57.71	54.01012
